# The complete chloroplast genome of the aromatic tree species *Cinnamomum tenuipile* Kosterm (Lauraceae)

**DOI:** 10.1080/23802359.2021.1997119

**Published:** 2022-02-03

**Authors:** Yongjie Zheng, YanFang Wu, Liu Xinliang, Yicun Chen, YangDong Wang

**Affiliations:** a Research Institute of Subtropical Forestry, Chinese Academy of Forestry, Hangzhou, China; b Jiangxi Academy of Forestry, Nanchang, China

**Keywords:** Chloroplast genome Lauraceae, *Cinnamomum tenuipile*, phylogenetic analysis

## Abstract

*Cinnamomum tenuipile* Kosterm is a precious aromatic tree in Lauraceae. To better determine its phylogenetic location with other *Cinnamomum* species, the chloroplast genome of *C. tenuipile* was sequenced. The complete chloroplast genome size is 152,761 bp, consisting of a pair of inverted repeats (IRa/b) with a length of 20,074 bp separated by a large single-copy region (LSC) and a small single-copy region (SSC) which are 93,685 and 18,928 bp, respectively. The overall GC content of the cp genome is 39.16%. The maximum-likelihood phylogenetic tree showed that *C. tenuipile* is more closely related to *C. aromaticum*, providing new insight into the evolution of Lauraceae.

*Cinnamomum tenuipile* is an aromatic tree species in Lauraceae that native to southwestern China. The essential oils distilled from *C. tenuipile* contain 86–98% geraniol, which has economic importance in the spice industry (Yang et al. 2005). However, excessive logging leads to the reduction of natural resources of *C. tenuipile*. In order to better protect and understand the phylogeny of *C. tenuipile*, the chloroplast genome has been studied based on high-throughput sequencing approaches.

DNA was isolated from fresh young leaves of *C. tenuipile* at Xishuangbanna (21.69°N,100.06°E). The voucher specimen (ZSS-ZYJ-20200830) was deposited at Camphor Engineering Technology Research Center for National Forestry and Grassland Administration (Yongjie Zheng, zyj920581676@gmail.com). A 350 bp library was constructed and sequenced by using the Illumina NovaSeq platform. Raw reads were polished and assembled by SPAdes (version: 3.13.0) (Bankevich et al. 2012) with the reference genome sequence of *Cinnamomum camphora* (GenBank: NC_035882) (Chen et al. 2017). The complete chloroplast (cp) genome sequence has been published on the GenBank with accession number: MW421304.

The cp genome size of *C. tenuipile* was 152,761 bp, including an LSC region of 93,685 bp, an SSC region of 18,928 bp, and a pair of IR regions of 20,074 bp. The overall GC content is 39.14% (LSC, 37.96%; SSC, 33.91%; IR, 44.44%). The cp genomes were annotated with 131 genes, including 83 protein-coding genes, 44 tRNA genes, and 4 rRNA genes.

A phylogenetic tree was reconstructed to confirm the phylogenetic location of *C. tenuipile*, with *Laurus* species as an outgroup (Figure 1). The complete chloroplast genome sequences were aligned by using MAFFT (Katoh et al. 2002). Maximum likelihood (ML) phylogenetic analyses were performed based on K3Pu + F + I + G4 model by ModelFinder and iqtree (version:1.6.7) with 1000 bootstrap replicates (Nguyen et al. 2015; Kalyaanamoorthy et al. 2017; Hoang et al. 2018). The ML phylogenetic tree with 83%–100% bootstrap values supported that *C. tenuipile* and *C. aromaticum* were in the same clade. The complete chloroplast genome of *C. tenuipile* provides valuable genomic resources for improving our understanding of species phylogeny in Lauraceae, exploring genetic variations and designing conservation strategies.

**Figure 1.**
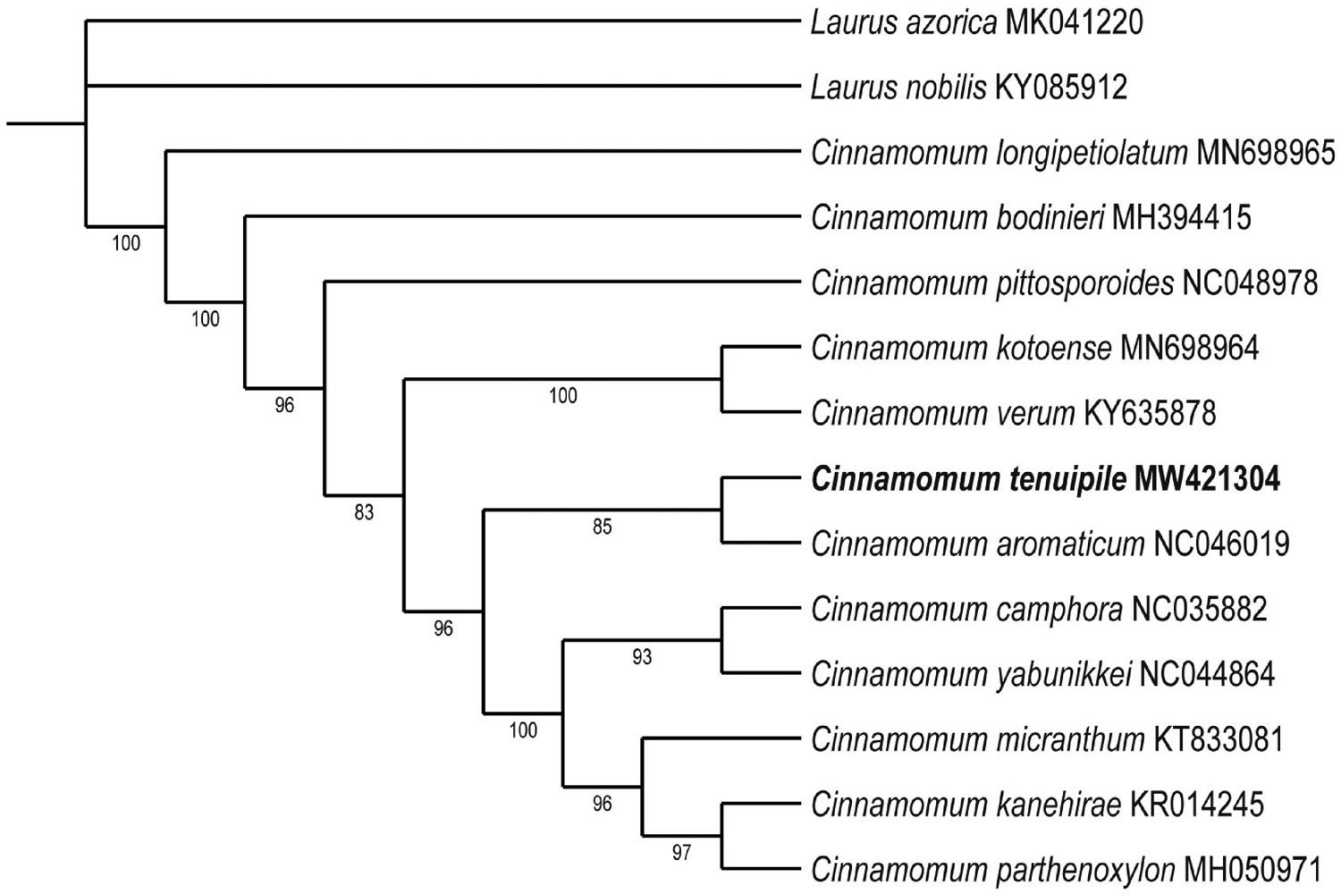
The ML phylogenetic tree for *C. tenuipile* based on the complete chloroplast genome sequences. The *Laurus* species was set as an outgroup.

## Data Availability

The genome sequence data that support the findings of this study are openly available in GenBank of NCBI at [https://www.ncbi.nlm.nih.gov] (https://www.ncbi.nlm.nih.gov/) under the accession no. MW421304. The associated BioProject, SRA, and Bio-Sample numbers are PRJNA749606, SAMN20394009, and SRR15243023, respectively.

## References

[CIT0001] Bankevich A, Nurk S, Antipov D, Gurevich AA, Dvorkin M, Kulikov AS, Lesin VM, Nikolenko SI, Pham S, Prjibelski AD, et al. 2012. SPAdes: a new genome assembly algorithm and its applications to single-cell sequencing. J Comput Biol. 19(5):455–477. 22506599 10.1089/cmb.2012.0021 PMC3342519

[CIT0002] Chen C, Zheng Y, Liu S, Zhong Y, Wu Y, Li J, Xu L-A, Xu M. 2017. The complete chloroplast genome of *Cinnamomum camphora* and its comparison with related Lauraceae species. PeerJ. 5:e3820. 28948105 10.7717/peerj.3820 PMC5609524

[CIT0003] Hoang DT, Chernomor O, von Haeseler A, Minh BQ, Vinh LS. 2018. UFBoot2: improving the ultrafast bootstrap approximation. Mol Biol Evol. 35(2):518–522. 29077904 10.1093/molbev/msx281 PMC5850222

[CIT0004] Kalyaanamoorthy S, Minh BQ, Wong TKF, von Haeseler A, Jermiin LS. 2017. ModelFinder: fast model selection for accurate phylogenetic estimates. Nat Methods. 14(6):587–589. 28481363 10.1038/nmeth.4285 PMC5453245

[CIT0005] Katoh K, Misawa K, Kuma K, Miyata T. 2002. MAFFT: a novel method for rapid multiple sequence alignment based on fast Fourier transform. Nucleic Acids Res. 30(14):3059–3066. 12136088 10.1093/nar/gkf436 PMC135756

[CIT0006] Nguyen L-T, Schmidt HA, von Haeseler A, Minh BQ. 2015. IQ-TREE: a fast and effective stochastic algorithm for estimating maximum-likelihood phylogenies. Mol Biol Evol. 32(1):268–274. 25371430 10.1093/molbev/msu300 PMC4271533

[CIT0007] Yang T, Li J, Wang HX, Zeng Y. 2005. A geraniol-synthase gene from *Cinnamomum tenuipilum*. Phytochemistry. 66(3):285–293. 15680985 10.1016/j.phytochem.2004.12.004

